# The Role of Economic Stress, Health Concerns, and Institutional Trust in Supporting Public Protests against COVID-19 Lockdown Measures in Denmark

**DOI:** 10.3390/ijerph20010148

**Published:** 2022-12-22

**Authors:** Jens Fyhn Lykke Sørensen, Maiken Christiansen

**Affiliations:** Department of Sociology, Environmental and Business Economics, University of Southern Denmark, Degnevej 14, 6705 Esbjerg Ø, Denmark

**Keywords:** COVID-19 lockdown, attitudes, public protests, socio-economic factors, urbanization, health concerns, institutional trust, incidence rates, ordered logit regression, structural equation modeling (SEM), Denmark

## Abstract

During the current COVID-19 pandemic, most governments around the world have adopted strict COVID-19 lockdown measures. In Denmark, mainly from January to March 2021, an anonymous protest group called Men in Black organized demonstrations against the Danish COVID-19 lockdown measures in the three major cities in Denmark. Based on an online survey that we carried out in March 2021 in the Danish population aged 16 years and above (*n* = 2692), we analyze the individual-level factors behind supporting these demonstrations. Based on ordered logit regressions, the results show that being Muslim and being self-employed (business owner) was positively related to supporting the demonstrations, and that age and living in a city municipality was negatively related to supporting the demonstrations. Based on structural equation modeling (SEM), the results showed that the municipal COVID-19 incidence rate mediates the effect of living in a city municipality, that institutional trust mediates the effect of being Muslim, and that COVID-19 health concerns and institutional trust mediate the effect of age. Overall, economic stress among business owners, health concerns, and institutional trust were found to be the main predictors of supporting the demonstrations against the COVID-19 lockdown measures in Denmark.

## 1. Introduction

During the current COVID-19 pandemic, most governments around the world have adopted strict anti-COVID-19 measures to curb the spread of the coronavirus (SARS-CoV-2 virus). Among the measures have been actual lockdown of societies involving measures such as assembly bans, shutdown of businesses, shutdown of day care, schools and other public institutions, ordering people to work from home, etc.

Obviously, the effectiveness of such measures also depends on whether citizens accept them and abide by them. This has not fully been the case as evidenced by demonstrations against COVID-19 lockdown measures in several countries [1,2]. The aim of this paper is to analyze the individual-level factors behind supporting such demonstrations based on the Danish case.

Denmark was one of the first European countries to introduce lockdown measures, starting on 11 March 2020. The lockdown formally ended in September 2021. At various stages of the lockdown, you would find the following restrictions: A ban to travel abroad, social isolation in case of infection, assembly bans, a ban on visiting vulnerable family members, the shutdown of daycare, the shutdown of all educational and sports facilities, the shutdown of shopping malls, shops and retail businesses (except supermarkets and pharmacies), restaurants, cafes, pubs, and discotheques, and the ordering of public employees in most public institutions to work from home.

All the lockdown measures were supported by a huge majority of the Danish parliament. In an international comparison, the Danish government introduced strict and quick lockdown measures and has been successful in curbing COVID-19 morbidity and excess mortality [3]. Denmark’s neighboring country, Sweden, for example, followed far less strict COVID-19 policies by relying more on voluntary, rather than mandatory, measures. In turn, based on counterfactual calculations, a study found find that “if Denmark had adopted Swedish policies, and introduced them at the same stage of its epidemic, mortality would have been between three and four times higher” (p. 5) [4].

In Denmark, as a reaction to the strict lockdown measures, a group of people formed an anonymous protest group called Men in Black in December 2020 [5]. In January 2021 and the following months, the group organized demonstrations against the COVID-19 lockdown measures in the three major cities in Denmark [6,7]. The Men in Black demonstrations received a lot of media attention and led to a substantial discussion among the public.

In this paper, we analyze the public attitudes towards these demonstrations based on data from a survey that we carried out in March 2021 in the Danish population among individuals aged 16 years and above (*n* = 2692). In assessing who may support demonstrations against COVID-19 lockdown measures, the first attention turns to various socioeconomic factors. Since the COVID-19 mortality rate is increasing with age [8], the acceptance of COVID-19 lockdown measures is expected to increase with age, and thus the support for demonstrations is likely to decrease with age. Moreover, the more people suffer financially under the COVID-19 lockdown measures, the more likely they are to support the demonstrations. Thus, people who have been laid off because of the COVID-19 lockdown and business owners who have lost their business or a good portion of their revenues due to the lockdown may all be more supportive of the demonstrations than other groups.

A potentially important, but rather overlooked, likely factor is the degree of urbanization. Thus, as the distance between people decreases with urbanization, the risk of getting infected through airborne transmission of the COVID-19 virus increases with urbanization [9,10,11]. Therefore, faced with the increased risk of getting infected, people in very urbanized areas may be more accepting of COVID-19 lockdown measures than people who live in less densely or rural areas.

With regard to urbanization, yet another factor may play a role. Recently, in certain cases in the Western world, an anti-system and populist sentiment has been found to exist among residents in non-metropolitan and rural areas [12,13,14]. For people living in non-metropolitan and rural areas, therefore, there might be two forces pulling in the same direction. First, the lower risk of getting infected may lower the acceptance of the COVID-19 lockdown measures, and second, an anti-system sentiment would also lower the acceptance of the COVID-19 lockdown measures.

Two other related factors are likely to be relevant: Institutional trust and the local COVID-19 incidence rate. The acceptance of COVID-19 lockdown measures is likely to depend on the level of trust towards the government and other public institutions. If you do not trust your government to make wise decision, and if you do not trust public institutions to do a good job, you are less accepting towards a governmental decision to introduce COVID-19 lockdown measures. Therefore, the likelihood of supporting the demonstrations against the COVID-19 lockdown measures in Denmark is likely to decrease with the level of institutional trust. Obviously, the local infection rate may mediate people’s attitude towards accepting COVID-19 lockdown measures. People in places where the COVID-19 incidence rate is high are more likely to accept them than people who live in places with low incidence rates. Apart from being interesting to study in their own right, including institutional trust and the local COVID-19 incidence rate has the additional advantage that it will enable us to explore the underlying mechanisms behind the effect of urbanization.

This paper will contribute to a small but growing literature on the subject. Thus, a few previous studies have analyzed the public attitudes towards COVID-19 lockdown measures in the following countries: Malaysia and India [15], Italy [16], France [17], Spain [18], Greece [19], and a collection of EU countries [20]. In this literature, the following socioeconomic factors were found to be positively linked to accepting lockdown measures: being male and having a medical background [15], being a female [18], having a high education [17,18,19], having a high income [17,20], and being materially non-deprived [19]. Moreover, people who were concerned with their own health [18,19,20] and people with high trust in public institutions [16,19] were more likely to support the lockdown policies. This branch of literature could be referred to as the ‘satisfaction’ literature.

A related branch of literature is about predicting individual compliance with COVID-19 measures [21,22,23,24,25]. This ‘compliance’ literature found some of the same factors as the ‘satisfaction’ literature, e.g., a significant effect of institutional trust [21,23,24]. However, the two branches of literature are intrinsically different since compliance and satisfaction do not necessarily go hand in hand. For example, to avoid fines or criminal charges, business owners are likely to comply with a law that requires them to shut down their private businesses. However, most business owners are likely to be dissatisfied with having to shut down their businesses because of revenue losses.

We offer four contributions to the existing ‘satisfaction’ literature. First, we provide evidence from a country that has not been studied before. Two, we explore attitudes towards demonstrations against COVID-19 lockdown measures. Looking specifically at attitudes towards demonstrations against COVID-19 lockdown measures is unprecedented. Third, we add to the literature by analyzing the potential role of urbanization. We will analyze whether attitudes in non-metropolitan and rural areas are driven by low COVID-19 infection rates or by anti-system sentiments in these areas. Forth and finally, we include additional and potentially important factors that have not been included in previous studies, e.g., economic factors, religious denomination, and local COVID-19 incidence rates.

## 2. Methods

### 2.1. Case: COVID-19 Lockdown Measures and Public Protests against Them in Denmark

On 26 February 2020, Denmark had it first confirmed case of COVID-19. On 11 March, after sharp increases in COVID-19 cases, the Danish government decided to shut down the country through several restrictive measures [26]. To use another term, the Danish government introduced a lockdown. Lockdown is the umbrella term for shutting down or restricting access to certain activities. The purpose of the lockdown was to curb the spread of the coronavirus by limiting the physical contact between people.

The basic elements in the Danish lockdown included a ban to travel abroad, social isolation in case of infection, assembly bans, and a ban on visiting vulnerable family members. Specifically, indoor visits to family members who stayed in nursing homes was banned [27]. Further, the lockdown included the shutdown of daycare, the shutdown of all educational and sports facilities, and the shutdown of shopping malls, shops and retail businesses (except supermarkets and pharmacies), restaurants, cafes, pubs, and discotheques. Universities were physically closed, but teaching continued online. Public institutions other than health institutions were also shut down in the sense that they were physically closed to the public, while people employed in the local, regional, and state administration had to work from home [26,28].

On 15 April 2021, the Danish government reopened daycare and permitted younger school children go back to school. Later, in May 2021, all students in the primary and secondary school system were allowed to return to school, whereas university students first returned to the university on 1 August 2021. Since the lockdown had had serious economic consequences for private companies, almost all the bans on private companies were removed quite early throughout May 2021. Later, employees in public institutions were allowed to physically return to their workplaces, and by September 2021, most lockdown measures had come to an end [26].

As mentioned, in January 2021 and the following months, an anonymous protest group called Men in Black organized demonstrations against the lockdown measures in the three major cities in Denmark. The purpose of the demonstrations was to utter strong dissatisfaction with having to live under the lockdown that was introduced by Danish government to fight the COVID-19 pandemic in Denmark [5,6,7]. For example, the group stated on their Facebook page: “We have had it with the arbitrary lockdown of the country” [7]. Additionally, the following slogan was repeatedly shouted out during the protests: “Freedom for Denmark, we have had enough [of the lockdown]!” [29].

**Figure 1:**
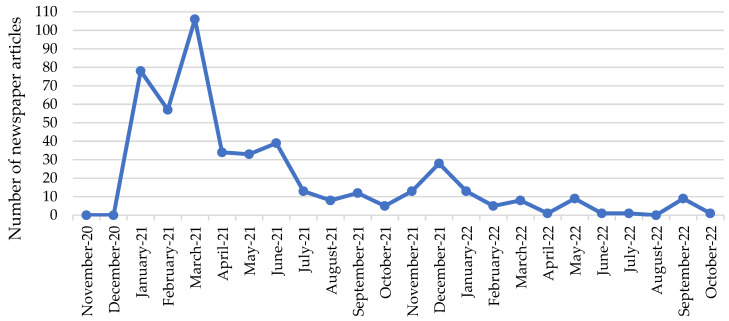
Number of newspaper articles mentioning Men in Black demonstrations in 10 Danish national newspapers, absolute number of articles per month, November 2020 to October 2022. Source: Infomedia. Note: Data includes the newspapers Avisen Danmark, B.T., Berlingske, Børsen, Ekstra Bladet, Information, Jyllands-Posten, Kristeligt Dagblad, Politiken, and Weekendavisen.

The Men in Black demonstrations received a lot of media attention. Thus, the demonstrations were reported widely in Danish television channels, and all national newspapers intensively covered the demonstration with news and background stories. Figure 1 shows the monthly number of newspaper articles reporting on the Men in Black demonstrations from November 2020 and onwards. As illustrated in the newspaper coverage in Figure 1, most demonstrations took place in the months of January, February, and March 2021. The small peak in newspaper reports in December 2021 is due to several trials that were carried out in that month against Men in Black demonstrators who had been arrested during the demonstrations earlier in 2021.

### 2.2. Data Collection

The analysis is based on data from an online survey that we carried out 8–16 March 2021 in the Danish population aged 16 years and above. The recruitment of respondents was done through convenience sampling by distributing the survey through various Facebook groups.

We chose to distribute the survey through Facebook for convenience and speedy collection of data and because Facebook is the most used social media in Denmark. Thus, in 2019, 77% of the Danish population had a Facebook profile, which was almost twice as much as the runners-up LinkedIn, Instagram, and Snapchat (40–41%). Moreover, in 2019, 64% of the Danish population used Facebook daily or several times daily, whereas 7%, 25% and 24% of the Danish population used LinkedIn, Instagram, and Snapchat daily or several times daily [30]. In selecting Facebook groups, we aimed for a well-balanced selection, that is, where we could assume that the people visiting the Facebook groups would provide a reasonable varied sample of the Danish population, which in turn would enable us to analyze intergroup differences. Table 1 shows examples of names of the Facebook groups that we used. The total sample consists of 3834 individuals, aged 16 to 85. After correcting for missing values, the sample was reduced to 2692 individuals, see Table A1 in the Appendix A.

### 2.3. Measurements

The questionnaire consisted of 49 questions, including nine personal background questions such as gender, age, education, etc. Most of the questionnaire dealt with people’s attitudes towards getting a COVID-19 vaccine. However, the questionnaire had one single question regarding people’s attitude towards the demonstrations against the COVID-19 lockdown measures of the Danish government that had taken place in the months prior to the survey. The attitude towards the demonstrations was measured by asking to what extent the respondent agrees in the following statement: “I support the demonstrations against the COVID-19 policies of the government”. There were five response options, and they were presented in the online questionnaire in the following order: Strongly disagree (coded 1), disagree (coded 2), neutral (coded 3), agree (coded 4), and strongly agree (coded 5). This variable will act as the dependent variable in our analyses.

We include the following socio-economic variables in the analysis: gender, age, civil status (living with partner/spouse or not), having children or not, religion, education, employment status, yearly gross income, and urbanization. In terms of urbanization, we use the so-called rural district classification that divides the 98 Danish municipalities into the following four municipality groups depending on the degree to which they contain rural areas: 16 peripheral municipalities, 29 rural municipalities, 18 intermediate municipalities, and 35 city municipalities [31]. Specifically, we include a dummy variable measuring whether the respondent lives in one of the 35 city municipalities or not.

To control for the local COVID-19 incidence rate, we use the official municipal incidence rate on new cases occurring in each week in Denmark. This municipal incidence rate is measured by the number of new COVID-19 cases per 100,000 inhabitants confirmed in each week in each municipality. Specifically, we use the weekly report of the test-adjusted incidence rates in each of Denmark’s 98 municipalities for the week 22 February to 1 March 2021 (Week 8). Incidence rates for this week were published on 5 March 2021 by Statens Serum Institut (SSI), which is the authority in Denmark under the Danish Ministry of Health that is responsible for ensuring preparedness against infectious diseases and biological threats. The incidence rates are published in publicly available PDF-files on the SSI homepage, and the incidence rates were frequently reported and discussed in the media, and we expect that the Danish population at the time of data collection had a good idea of the extent of the COVID-19 spread in their municipality.

We include emotional anxiety caused by the pandemic. We measure emotional anxiety by the following survey item: “How worried are about getting infected with COVID-19?”. The respondent had to answer on a scale from 1 to 10, where 1 meant ‘not worried’ to 10 meant ‘very worried’.

Inspired by the institutional trust items in the core module of the academically driven European Social Survey [32], our survey measures institutional trust in six items where the respondent must state his/her level of trust in the following: (1) The government, (2) The Prime Minister Mette Frederiksen, (3) The health authorities, (4) The healthcare system, (5) The police, and (6) The WHO (World Health Organization). The respondent had to answer on a scale from 0 to 10, where 0 meant ‘no trust whatsoever’ and 10 meant ‘very strong trust’. The combined institutional trust variable was created by adding the responses to the six items, thus getting the theoretical range of 0 to 60. The Cronbach’s *α* for the institutional trust variable was calculated to be 0.91, which is above the commonly used threshold of 0.7 [33].

In sum, we include a total of 13 variables in our analysis, 12 of which are derived from the survey. 11 of the 13 variables are categorical variables. Two are continuous variables: Age and the municipal COVID-19 incidence rate. There were no outliers attached to these two variables. Age ranged from 16 to 85 years. The average age was 41.5 years with a standard deviation of 14.7 years. The municipal COVID-19 incidence rate ranged from 0 to 226.5. The average municipal COVID-19 incidence rate was 50.2 with a standard deviation of 29.4. The incidence rate was equal to zero in the small municipality of Struer and in the four very small island municipalities of Ærø, Samsø, Fanø, and Læsø.

### 2.4. Statistical Methods

We will use two statistical methods that will supplement each other. The first method is ordered logit regression. Using this method, we will estimate the function:

*S_i_* = *α* + *βX_i_* + *ε* (1)

where *S_i_* is the reported support for the demonstrations of individual *i*, and *X_i_* is the vector of explanatory variables. The error term *ε* holds unobserved traits. Ordered logit estimation is used to estimate Equation (1), because the dependant variable—level of support for the demonstrations—is ordinally scaled [34].

To some degree, the local COVID-19 incidence rate, COVID-19 anxiety, and individual institutional trust could mediate the socioeconomic determinants of supporting the demonstrations. Therefore, we first estimate a reduced model that exclusively includes socioeconomic factors, then a model that adds the local COVID-19 incidence rate, and finally a model that adds COVID-19 anxiety and institutional trust. This will provide us with an idea of direct and mediating effects.

The second method is structural equation modeling (SEM) that we will use to test the direct and mediating effects suggested by the regression analyses. We will enter the only latent variable among the variables, institutional trust, as observed variable, that is, we will enter the combined institutional trust variable mentioned above. All statistical analyses were performed using SPSS 28 and Stata 17.

## 3. Results

### 3.1. Descriptive Statistics

Table 2 shows the attitudes toward the demonstrations against the COVID-19 lockdown measures. While most respondents are non-supportive of the demonstrations (69%), there are fractions of respondents who are neutral (19%), supportive (7%) or very supportive of the demonstrations (5%).

Table 3 shows descriptive statistics for the remaining variables as well as the distribution of attitudes toward the demonstrations within each variable stratum. As for descriptive statistics (column 3), judged by the gender variable alone, we can conclude that the sample is not representative of the study population (Danish population aged 16 and above). However, the sample distributions with regard to civil status, religion, education, employment status, and city municipality all lie close to the distributions within the study population. For example, as of 1 January 2021, 49.7% of the adult Danish population aged 16 and above lived in a city municipality. We calculated this percentage using publicly available statistics provided by Statistics Denmark, see www.statistikbanken.dk/BY2 (accessed on 19 November 2022). Moreover, we note that the municipal COVID-19 incidence rate variable as well as the COVID-19 anxiety variable and the institutional trust variable display a lot of variation. As for the distribution of attitudes toward the demonstrations (columns 4–6), people of the Muslim faith, self-employed people (business owners), those with low COVID-19 anxiety, and those with low institutional trust are the strongest supporters of the demonstrations, having support percentages ranging from 23% to 39% as compared to the overall support percentage of 12%.

### 3.2. Ordered Logit Regressions

In Table 4, we report the results of the three regression models. In Table 4, Model 1, we see the social stratification of supporting (or not supporting) the demonstrations. Self-employed people stand out as the employment group that supports the demonstrations the most. Moreover, Muslims are significantly more supportive of the demonstrations than those who do not belong to any denomination. Furthermore, people aged 60 years and above are significantly less supportive of the demonstrations. Finally, living in a city municipality is negatively related to supporting the demonstrations. This result was expected as people in cities are more at risk of getting infected than people living in less densely populated settings. Consequently, people in city municipalities would benefit more from nationwide COVID-19 lockdown measures than people in less densely populated places, and therefore people in city municipalities would be more inclined to accept the lockdown measures.

In Table 4, Model 2, the municipal COVID-19 incidence rate is added to Model 1. As expected, the municipal COVID-19 incidence rate is negatively related to supporting the demonstrations. Importantly, when adding the municipal COVID-19 incidence rate, the city municipality variable becomes statistically insignificant which underlines the above argument of why city municipality had a statistically significant, negative sign in Model 1. In other words, the municipal COVID-19 incidence rate seems to mediate the result for the city municipality variable in Model 1. Finally, we note that the results for the other significant variables in Model 1 remain unchanged in Model 2 when adding the municipal COVID-19 incidence rate.

In Table 4, Model 3, COVID-19 anxiety and institutional trust are added. As expected, COVID-19 anxiety as well as institutional trust are negatively related to supporting the demonstrations. Importantly, COVID-19 anxiety and/or institutional trust seem to mediate the effect of having the Muslim faith. Thus, when adding institutional trust, the effect of having the Muslim faith turns statistically insignifikant. Judged by the pairwise correlations, we suspect that institutional trust is the only mediator. Thus, as can be seen in the correlation matrix in Table A2 in the Appendix B, being Muslim is significantly negatively related to institutional trust, while there is no significant association between being Muslim and COVID-19 anxiety. To put it short, Muslims are less trusting towards public institutions, and this makes them more critical towards the COVID-19 lockdown measures. Furthermore, as age has turned insignificant in Model 3, COVID-19 anxiety and/or institutional trust seem to mediate the effect of age. We suspect that both COVID-19 anxiety and institutional trust could be mediators, since they are both significantly correlated with age, see correlation matrix in Table A2 in the Appendix B. Age is positive related to both COVID-19 anxiety and institutional trust. Therefore, the less supportive attitude towards the demonstrations of people in the oldest age group is likely to run through their higher COVID-19 anxiety and their higher institutional trust. Anxiety is higher because of the larger health risks for older people. Moreover, older people tend to trust public institutions more than younger people, and this may also be a reason for their higher non-support for the demonstrations.

The only socio-economic variable that remains strongly significant in Model 3 is the dummy variable for self-employed people. Meanwhile, some of the effect from being self-employed may be mediated by COVID-19 anxiety and institutional trust since the coefficient for self-employed drops from 1.0274 to 0.5923 from Model 2 to Model 3. From Table A2 in the Appendix B, we can see that being self-employed is negatively related to both institutional trust and COVID-19 anxiety. Hence, some of the supportive attitude towards the demonstrations may be explained by a lower COVID-19 anxiety and a lower institutional trust among self-employed people.

Next, we turn to the SEM analyses to further test the above assumptions about indirect and direct effects.

### 3.3. Structural Equation Modeling

We established a structural equation model (SEM) to examine the relationship between variables as hypothesized based on the ordered logit regressions. Thus, we included the variables that had significant effects in the regressions, while stipulating the pathways as discussed above. The model fit turned out to be acceptable. The goodness of fit indices are as follows: *χ*^2^ = 141.682, *p* < 0.001; comparative fit index (CFI) = 0.959; Tucker–Lewis index (TLI) = 0.886; standardized root mean square residual (SRMR) = 0.040; root mean squared error of approximation (RMSEA) = 0.079.

**Figure 2:**
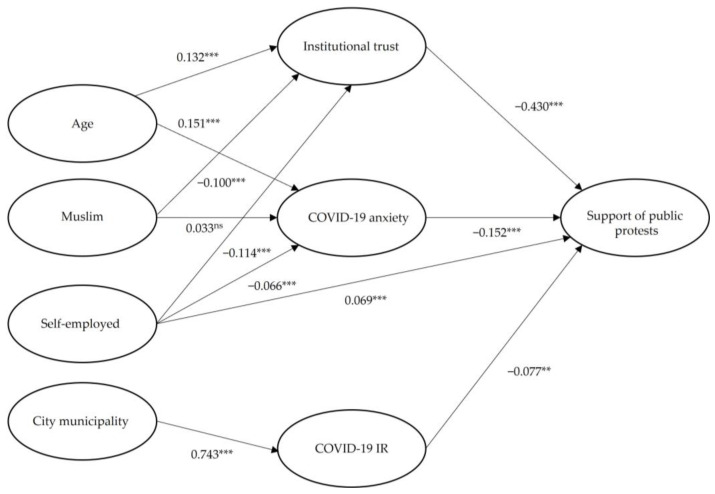
Structural equation model with standardized effects. Note: ** *p* < 0.01, *** *p* < 0.001, ns: not significant at the 5% significance level (two-tailed).

The model output is shown in Figure 2, displaying the correlations and effect paths of the variables. The model shows highly statistically significant direct effects of institutional trust, COVID-19 anxiety, COVID-19 incidence rate, and being self-employed. As expected, the total effect of being self-employed consists of the direct effect and an indirect effect that runs through both institutional trust and COVID-19 anxiety, cf. the significant correlations. Moreover, Figure 2 confirms that the lesser support of the demonstrations among people living in city municipalities is mediated by a greater COVID-19 incidence rate in city municipalities. As expected above, the greater support of the demonstration among people of the Muslim faith is mediated by a lower institutional trust in this group and not by a lower COVID-19 anxiety, cf. the significant negative coefficient (−0.100, *p* < 0.001) and the insignificant coefficient (0.033, *p* = 0.09), respectively. Finally, Figure 2 confirms our expectation that the lower support among older people is mediated by their higher COVID-19 anxiety and their higher institutional trust.

The evidence becomes clear in Table 5, showing the direct, indirect, and total effects. The variables age, Muslim, and city municipality only have indirect effects, and the variable self-employed has large direct as well as large indirect effects.

## 4. Discussion

In this paper, we investigated individual-level factors behind supporting the demonstrations against the Danish COVID-19 lockdown measures that took place in the beginning of 2021, based on a net sample of 2692 individuals drawn from the Danish population aged 16 and above in March 2021. We performed ordered logit regressions and an SEM analysis.

We performed three ordered logit estimations: the first included only socioeconomic variables, the second added the municipal COVID-19 incidence rate, and the third added COVID-19 anxiety and institutional trust.

The first model showed that being Muslim and being self-employed was positively related to supporting the demonstrations, whereas age and living in a city municipality was negatively related with supporting the demonstrations. Including the COVID-19 incidence rate at the municipality level in the second model mediated the effect of living in a city municipality. Accordingly, the result of more support for the demonstrations in non-metropolitan and rural areas does not reflect an anti-system or populist sentiment in these areas [12,13,14], but rather lower infection rates which makes strict COVID-19 lockdown measures seem less necessary. When including COVID-19 anxiety and institutional trust in the third and final model, age and having the Muslim faith lost all of their statistical significance. The SEM model showed that the effect of having the Muslim faith was mediated by institutional trust and that the effect of age was mediated by COVID-19 anxiety and institutional trust.

In the final model and in the SEM analysis, the group of self-employed people was the only socio-economic group with a direct effect. Several circumstances in Denmark can explain why self-employed people were uniquely supportive of the demonstrations against the COVID-19 lockdown measures in Denmark. Hence, financially, it was much more critical being self-employed (business owner) than being a wage earner during the lockdown period in Denmark. Wage earners in public institutions could work from home or got sent home with full pay during the lockdown, and the government introduced financial help packages that contained money to wage earners in private firms. In the so-called Wage Compensation Package, business owners could apply for support that would cover 75–90% of their wage costs if they refrained from laying off employees [35]. Based on a survey conducted among 10,642 small, medium-sized and large Danish firms from 23 April to 1 June 2020, a study estimates that the help packages had saved up to 81,000 employees from being laid off in the first few months since the lockdown was introduced on 13 March 2020 [36]. According to our own calculations based on data found in the publicly available database of Statistics Denmark (www.statistikbanken.dk/AKU220K, accessed on 10 November 2022), 81,000 employees accounted for about 5% of the total number of people employed in the private sector in Denmark as of 1 April 2020. Self-employed people could also apply for support for themselves (to put into their own pockets) if they could document a turnover drop of at least 30%. In that case, they could apply for financial support that covered up to 90% of the reduction in turnover. However, this package stipulated a maximum monthly amount of 23,000 DKK per business owner [37], which is only a substantial amount to business owners who have a rather low turnover, e.g., self-employed individuals with a small business and no employees.

This paper investigated attitudes towards demonstrations against anti-COVID-19 measures in the Danish content. If we should venture to compare our evidence with the evidence from other countries, it seems that low-income and low-education groups in Denmark have been less financially challenged and thus have been much more supportive of the national COVID-19 lockdown measures than these groups have been in countries like France and Spain [17,18]. It would be interesting if future research could investigate this closer while considering cross-country differences in social security systems, the existence and content of help packages during the lockdowns, and the severity and the timing of the national lockdown measures.

The main limitation of this study is the use of convenience sampling which resulted in a sample that was not fully representative of the study population. It is however doubtful whether the results would have been different if a more representative sample had been used. The skewed sampling was mainly characterized by a strong overrepresentation of women. However, men and women did not seem to have different attitudes toward the demonstrations against the COVID-19 measures, as the results showed. Another limitation is the fact that we only had one survey item that measured the attitude toward the demonstrations. To examine validity issues, we should have included several different survey items that measure the attitudes toward the demonstrations and the COVID-19 lockdown of the Danish government. Moreover, the survey item we did use was not standardized and perhaps not the most straightforward. However, during the nine days while the survey was running, the demonstrations were very high on the news agenda in Denmark, and we do not think that respondents found the question hard to relate to nor hard to answer. Nevertheless, we cannot rule out some misclassification bias, and future studies are urged to be attentive towards this issue.

## 5. Conclusions

Based on an online survey that we carried out in March 2021 in the Danish population aged 16 years and above (*n* = 2692), we analyzed the individual-level factors behind supporting these demonstrations. Based on ordered logit regressions, the results show that being Muslim and being self-employed (business owner) was positively related to supporting the demonstrations, and that age and living in a city municipality was negatively related to supporting the demonstrations. Based on structural equation modeling (SEM), the results showed that the municipal COVID-19 incidence rate mediates the effect of living in a city municipality, that institutional trust mediates the effect of being Muslim, and that COVID-19 health concerns and institutional trust mediate the effect of age. Overall, economic stress among business owners, health concerns, and institutional trust were found to be the main predictors of supporting the demonstrations against the COVID-19 lockdown measures in Denmark.

## Figures and Tables

**Table 1:** Examples of Facebook groups used for recruiting respondents in March 2021.

Facebook Group Name (Danish)	Facebook Group Name (English Translation)
Friluftsliv	Outdoor life
Info fra borger til borger i Esbjerg	Info from citizen to citizen in Esbjerg
Venligboerne København og omegn	Kind dwellers in Copenhagen and surroundings
Salg Odense og Fyn	Sales Odense and Funen
Roskilde hjælper hinanden	Roskilde is helping each other
Odense	Odense
Hjælp hinanden	Help each other
Kolding	Kolding
Studerende i Odense//students in Odense	Students in Odense
Hjælp hinanden i 2800 Lyngby og omegn	Help each other in 2800 Lyngby and surroundings
Job i Nordjylland,	Job in North Jutland
Markedspladsen Aarhus,	The Market Place Aarhus
Syddansk Universitet (SDU-Odense)	University of Southern Denmark (SDU-Odense)
Job søges-Job tilbydes	Seeking jobs-Offering jobs

Notes: Esbjerg, Copenhagen, Odense, Kolding, and Aarhus are large cities in Denmark. Lyngby is part of Copenhagen. Jutland is the large peninsular that borders Germany. Funen is a large island.

**Table 2:** Support of the demonstrations against the COVID-19 lockdown measures.

	Frequency, *n*	Frequency, %
Strong non-support of demonstrations	1338	50
Non-support of demonstrations	548	20
Neutral in relation to demonstrations	502	19
Support of demonstrations	179	7
Strong support of demonstrations	125	5
Total	2692	100

Note: Based on the survey item where we asked to what extent the respondent agrees in the following statement: “I support the public protests against the COVID-19 policies of the government”. There were five response options: strongly disagree, disagree, neutral, agree, strongly agree.

**Table 3:** Descriptive statistics and support of demonstrations by strata (*n* = 2692).

Variable	Variable Categories	Descriptive Statistics, %	Support of Demonstrations, %		
			Non-Support	Neutral	Support
Gender	Male	21	70	18	12
	Female	79	70	19	11
Age	16–29 years old	29	67	23	9
	30–39 years old	16	68	22	10
	40–49 years old	22	71	17	13
	50–59 years old	21	71	16	13
	60+ years old	12	77	12	11
Partner	Has no partner	27	70	20	10
	Has a partner	73	70	18	12
Children	Has no children	38	68	22	10
	Has child(ren)	62	71	17	12
Religion	Has no religion	38	70	19	11
	Christian	55	71	18	11
	Muslim	2	45	32	23
	Other religion	5	72	18	10
Education	Primary school	6	61	30	9
	Secondary school	17	67	23	10
	Vocational education	21	70	16	14
	Higher education, short	9	69	18	13
	Higher education, medium	33	72	18	10
	Higher education, long	14	75	14	11
Employment	Wage earner	55	72	17	11
	Self-employed	5	49	23	28
	Unemployed	9	67	25	9
	Outside workforce	31	71	19	10
Income	Less than DKK 150,000	27	66	25	10
	DKK 150,000–399,999	48	72	17	11
	DKK 400,000 or more	24	71	15	14
Degree of urbanization	Not living in a city municipality	48	68	19	12
	Living in a city municipality	52	72	18	10
COVID-19 incidence rate (IR) in municipality	0–19.99	13	64	21	14
	20–39.99	32	70	18	12
	40–59.99	16	66	23	11
	60–79.99	10	72	16	12
	80+	30	74	16	9
COVID-19 anxiety	1–2	21	52	24	25
	3–4	24	69	22	9
	5–6	23	72	18	9
	7–8	23	81	13	5
	9–10	9	80	13	7
Institutional trust	0–29.99	13	25	36	39
	30–39.99	20	54	34	12
	40–49.99	36	79	15	6
	50+	31	89	6	5

Note: Based on the survey item where we asked to what extent the respondent agrees in the following statement: “I support the public protests against the COVID-19 policies of the government”. There were five response options: strongly disagree, disagree, neutral, agree, strongly agree. ‘Non-support’ includes the first two options, and ‘Support’ includes the two last options.

**Table 4:** Support of demonstrations against the COVID-19 lockdown measures. Ordered logit regression.

Variable	Variable Categories	Support	Model 1		Model 2		Model 3	
		%	Coefficient	*z*-Value	Coefficient	*z*-Value	Coefficient	*z*-Value
Gender	Male (reference)	12						
	Female	11	−0.0555	−0.59	−0.0600	−0.64	0.1210	1.22
Age	16–29 years old (reference)	9						
	30–39 years old	10	−0.1279	−0.95	−0.1322	−0.98	−0.1718	−1.22
	40–49 years old	13	−0.2386	−1.68	−0.2485	−1.74	−0.1211	−0.81
	50–59 years old	13	−0.2881	−1.94	−0.2933 *	−1.97	−0.1009	−0.65
	60+ years old	11	−0.5940 ***	−3.57	−0.5849 ***	−3.50	−0.2222	−1.27
Partner	Has no partner (reference)	10						
	Has a partner	12	0.0219	0.26	0.0157	0.19	0.0851	0.96
Children	Has no children (reference)	10						
	Has child(ren)	12	0.0073	0.07	0.0027	0.02	0.0432	0.37
Religion	Has no religion (reference)	11						
	Christian	11	−0.0776	−0.98	−0.0923	−1.16	0.1184	1.41
	Muslim	23	0.8474 **	3.03	0.8789 **	3.13	0.3823	1.31
	Other religion	10	−0.0587	−0.36	−0.0452	−0.28	0.0582	0.34
Education	Primary school (reference)	9						
	Secondary school	10	−0.2237	−1.33	−0.2385	−1.41	0.0064	0.04
	Vocational education	14	−0.1608	−0.97	−0.1662	−1.00	−0.0187	−0.11
	Higher education, short	13	−0.0259	−0.14	−0.0164	−0.09	0.0124	0.06
	Higher education, medium	10	−0.2485	−1.56	−0.2385	−1.50	−0.0180	−0.11
	Higher education, long	11	−0.3389	−1.86	−0.3028	−1.66	0.0330	0.17
Employment	Wage earner (reference)	11						
	Self-employed	28	1.0201 ***	5.64	1.0274 ***	5.66	0.5923 **	3.27
	Unemployed	9	0.1075	0.79	0.0996	0.73	0.0935	0.65
	Outside workforce	10	−0.0346	−0.33	−0.0191	−0.18	−0.0086	−0.08
Income	DKK 150,000 (reference)	10						
	DKK 150,000–399,999	11	−0.1497	−1.32	−0.1504	−1.32	−0.1307	−1.11
	DKK 400,000 or more	14	−0.1220	−0.83	−0.1168	−0.79	−0.1557	−1.01
Degree of urbanization	Not living in a city municipality (reference)	12						
	Living in a city municipality	10	−0.1825 *	−2.38	−0.0027	−0.02	0.0166	0.13
COVID-19 incidence rate (IR) in municipality	0–19.99 (reference)	14						
	20–39.99	12			−0.1479	−1.22	−0.0004	−0.00
	40–59.99	11			−0.1259	−0.80	0.0141	0.09
	60–79.99	12			−0.2549	−1.40	−0.0842	−0.44
	80+	9			−0.4414 **	−2.74	−0.3546 *	−2.12
COVID-19 anxiety	1–2	25						
	3–4	9					−0.5895 ***	−5.32
	5–6	9					−0.5491 ***	−4.78
	7–8	5					−1.0942 ***	−9.12
	9–10	7					−1.0065 ***	−6.28
Institutional trust score	0–29.99 (reference)	39						
	30–39.99	12					−1.1183 ***	−8.76
	40–49.99	6					−2.0489 ***	−16.44
	50+	5					−2.8931 ***	−21.03
Number of observations			2692		2692		2692	
Pseudo *R*^2^ (Nagelkerke)			0.033		0.037		0.284	

Note: The dependent variable in the ordered logit regressions draws on the following survey item: Do you agree in the following statement? “I support the demonstrations against the COVID-19 policies of the government”. Response options: strongly disagree (coded 1), disagree (coded 2), neutral (coded 3), agree (coded 4) and strongly agree (coded 5). The second column titled “Support” shows the percentage who answered either “agree” or “strongly agree”. * *p* < 0.05, ** *p* < 0.01, *** *p* < 0.001 (two-tailed).

**Table 5:** Direct, indirect, and total effects, coefficients.

	Direct Effects	Indirect Effects	Total Effects
Age	0.0069	0.0659 ***	0.0590 ***
Muslim	0.2887	0.3461 ***	0.6348 ***
Self-employed	0.3780 ***	0.3245 ***	0.7025 ***
City municipality	0.0315	−1.1320 **	−0.1005 *
COIVD-19 IR	−0.0611 **		−0.0611 **
COVID-19 anxiety	−0.1379 ***		−0.1379 ***
Institutional trust	−0.4902 ***		−0.4902 ***

* *p* < 0.05, ** *p* < 0.01, *** *p* < 0.001 (two-tailed).

## Data Availability

The data used in this study is available upon request from the corresponding author.

## Appendix A

**Table A1:** Degree of completion of questionnaire items. Gross sample: *n* = 3834.

Questionnaire Items	Number of Responses	Number of Missing Values	Degree of Completion, %
Support of demonstrations	3829	5	99.9
Gender	3798	36	99.1
Age	3828	6	99.8
Partner	3817	17	99.6
Children	3765	69	98.2
Religion	3645	189	95.1
Education	3746	88	97.7
Employment	3813	21	99.5
Income	3722	112	97.1
Degree of urbanization	3803	31	99.2
COVID-19 anxiety	3205	625	83.6
Institutional trust	3565	269	93.0

Note: Net sample = 2692. The net sample consists of the respondents who have given a response to all 12 questions.

## Appendix B

**Table A2:** Correlation matrix (*n* = 2692).

Variables	1	2	3	4	5	6	7	8	9	10	11	12	13	14	15	16	17	18	19	20	21	22	23
1 Support of demonstrations	1																						
2 Female	−0.01	1																					
3 Age	**−0.06**	**−0.04**	1																				
4 Has partner	0.00	0.01	**0.09**	1																			
5 Has children	−0.03	**0.06**	**0.63**	**0.23**	1																		
6 Has no religion	0.01	**−0.08**	**−0.18**	**−0.09**	**−0.16**	1																	
7 Christian	−0.03	**0.08**	**0.22**	**0.12**	**0.20**	**−0.86**	1																
8 Muslim	**0.07**	0.02	**−0.10**	−0.02	**−0.06**	**−0.10**	**−0.14**	1															
9 Other religion	−0.00	−0.02	**−0.05**	**−0.05**	**−0.08**	**−0.19**	**−0.26**	−0.03	1														
10 Primary school	0.03	−0.00	**−0.04**	−*0.04*	**−0.06**	−0.00	−0.01	−0.01	*0.03*	1													
11 Secondary school	0.02	0.02	**−0.38**	**−0.11**	**−0.37**	**0.06**	**−0.08**	**0.04**	0.03	**−0.12**	1												
12 Vocational education	0.01	−0.01	**0.16**	**0.04**	**0.18**	**−0.10**	**0.13**	**−0.06**	−*0.03*	**−0.13**	**−0.23**	1											
13 Higher education, short	0.02	−0.02	**0.12**	0.01	**0.07**	−*0.03*	**0.05**	−0.02	−*0.04*	**−0.08**	**−0.14**	**−0.17**	1										
14 High. education, medium	−0.03	**0.06**	**0.12**	*0.04*	**0.13**	−0.00	0.00	0.02	−0.00	**−0.18**	**−0.31**	**−0.37**	**−0.22**	1									
15 Higher education, long	−0.03	**−0.07**	−0.02	*0.04*	−0.01	**0.09**	**−0.11**	0.03	0.02	**−0.10**	**−0.18**	**−0.21**	**−0.13**	**−0.28**	1								
16 Wage earner	**−0.05**	−0.01	**0.17**	**0.11**	**0.28**	**−0.04**	**0.08**	**−0.06**	**−0.06**	**−0.14**	**−0.23**	**0.06**	**0.06**	**0.09**	**0.10**	1							
17 Self-employed	**0.12**	**−0.07**	**0.11**	**0.04**	**0.08**	−0.02	0.00	−0.00	0.03	−0.02	−*0.04*	*0.04*	−0.01	−0.02	**0.05**	**−0.24**	1						
18 Unemployed	0.01	**0.05**	**−0.07**	**−0.04**	−*0.03*	0.00	−0.03	0.02	**0.04**	**0.08**	**−0.05**	0.02	−0.00	−0.02	0.01	**−0.35**	**−0.07**	1					
19 Outside workforce	−0.01	0.01	**−0.19**	**−0.11**	**−0.31**	**0.05**	**−0.08**	**0.05**	0.02	**0.11**	**0.30**	**−0.09**	**−0.06**	**−0.07**	**−0.14**	**−0.75**	**−0.15**	**−0.21**	1				
20 Income	−0.03	**−0.19**	**0.45**	**0.13**	**0.43**	**−0.06**	**0.10**	**−0.07**	**−0.05**	**−0.17**	**−0.36**	**0.06**	**0.10**	**0.10**	**0.22**	**0.53**	**0.11**	**−0.16**	**−0.51**	1			
21 City municipality	−*0.04*	**−0.07**	**−0.13**	**−0.07**	**−0.17**	**0.15**	**−0.17**	**0.04**	0.02	**−0.07**	**0.08**	**−0.14**	−0.03	−0.02	**0.17**	**−0.08**	*0.04*	−0.02	**0.08**	−*0.03*	1		
22 COVID-19 incidence rate	**−0.07**	**−0.07**	**−0.10**	**−0.08**	**−0.16**	**0.15**	**−0.18**	**0.06**	**0.04**	**−0.05**	**0.04**	**−0.14**	−0.02	−0.01	**0.19**	**−0.07**	*0.03*	−0.03	**0.08**	−0.03	**0.74**	1	
23 COVID-19 anxiety	**−0.25**	**0.13**	**0.14**	0.00	**0.12**	**−0.10**	**0.09**	0.02	0.00	−0.02	**−0.05**	**0.09**	−0.01	−0.01	−0.02	−0.03	**−0.05**	**0.05**	0.03	−0.02	**−0.04**	−*0.03*	1
24 Institutional Trust	**−0.47**	0.03	**0.13**	**0.06**	**0.09**	**−0.08**	**0.12**	**−0.11**	−0.03	**−0.05**	−*0.03*	−0.01	**−0.05**	**0.06**	0.03	**0.08**	**−0.10**	−*0.03*	−0.02	**0.06**	0.01	0.02	**0.23**

Notes: In bold: Significant at the 5% level. In italics: Significant at the 10% level. Supporting demonstrations = 5-scale categorical variable as used in Table 4. Age, income, COVID-19 incidence rate and institutional trust entered as same categorical variables as in Table 4.
